# Seminal papers in urology: urinary volume, water and recurrences in idiopathic calcium nephrolithiasis: a 5-year randomized prospective study: Borghi L, Meschi T, Amato F, Briganti A, Novarini A, Giannini A. urinary volume, water and recurrences in idiopathic calcium nephrolithiasis: a 5-year randomized prospective study. The Journal of urology. 1996 Mar;155(3):839 − 43

**DOI:** 10.1186/s12894-024-01416-9

**Published:** 2024-02-03

**Authors:** Adam Collingridge, Michael O’Callaghan

**Affiliations:** 1 Urology Unit, Flinders Medical Centre, Adelaide, Australia; 2 Discipline of Medicine, University of Adelaide, Adelaide, Australia; 3 College of Medicine and Public Health, Freemasons Centre for Male Health and Wellbeing, Flinders University, Adelaide, Australia

**Keywords:** Calcium oxalate stone, Fluid intake, Nephrolithiasis, Randomized controlled trial, Urine volume

## Abstract

Kidney stones, a persistent urological condition, continue to affect people globally. In this critical review, we examine the work of Borghi et al. who evaluated patients with idiopathic stone formation and randomised 99 patients to increased water intake (≥ 2 L/day) and 100 patients to usual care in a 5-year randomized controlled trial. The study examined baseline urine volume in individuals with idiopathic calcium stones, recurrence rates, and relevant biochemical factors. The study found reduced recurrence rate (12.1% vs. 27% (p = 0.008)), and time to recurrence with increased water intake (38.7 ± 13.2 months) vs. (25 ± 16.4 months) (*p* = 0.016). These findings inform clinical practice, contributing to the guideline recommendations that kidney stone patients should aim for fluid intake of at least 2.5 L per day to prevent stone recurrence.

## Introduction

Kidney stones are common and have affected people throughout history [1]. Both incidence and prevalence vary globally due to factors such as geography, climate, age, sex, race, diet, and fluid intake [1]. They were first described in ancient Mesopotamian medical texts dating back to 3200 to 1200 BCE [1]. Famously, Hippocrates (460 − 377 BCE) discouraged physicians from ‘cutting for stone’ (performing cystotomies) in response to bladder stone pain due to the high risk of complications and death. Instead, he recommended that skilled lithotomists conduct the procedure using a perineal incision [2]. 

The research conducted by Borghi et al. between 1986 and 1996 addresses the issue of high recurrence rates of calcium-based stones within five years of the initial episode [3]. To comprehend the study’s significance, it is essential to consider both its historical context and its relevance in the present day, accounting for advancements in scientific methodology and changes in patient populations over the intervening years.

## Background and clinical guidelines

Before 1986, research on calcium-based stones revealed a recurrence rate of over 53% within five years [4]. Factors leading to calcium stone formation were categorised into low urine volume and mineral imbalances. To mitigate stone formation, researchers hypothesised that increasing urine volume could reduce the concentration of stone-forming salts. However, concerns were raised about the potential impact of increased water intake on calcium crystallisation inhibitors. Nevertheless, experiments conducted by Pak et al. provided further support for the idea that increased water intake could be beneficial. They conducted in vitro and in vivo experiments on kidney stone patients and controls, diluting urine by adding water. These experiments observed a reduced propensity for calcium salt crystallisation [5]. 

At present, both the American Urological Association (AUA) [6] and the European Association of Urology (EAU) Guidelines recommend a daily fluid intake of at least 2.5 L to prevent stone recurrence. These recommendations cite the study discussed in this paper.

## The paper’s objectives

Borghi et al. identified a lack of scientific evaluation regarding the preventive effectiveness of increased water intake. They aimed to address this gap and achieve three main objectives: (1) To quantify the baseline urine volume in individuals with idiopathic calcium stones; (2) To evaluate the preventive effectiveness of a simple high-water treatment (without dietary changes) in preventing stone recurrences and finally (3) to identify any biochemical urine factors that might predict stone recurrences. By conducting a thorough examination of these objectives, the study seeks to shed light on the potential benefits of increased water intake in preventing recurrent calcium-based kidney stones.

### Appraisal of study methods

The study followed a prospective randomized controlled trial design. Participants were randomly assigned to one of two follow-up programs: Program 1 involved a high-water intake (urine volume ≥ 2 L/day), while Program 2 did not include any high-water treatment, and were told it was not necessary to follow any special procedures. The primary outcome was the frequency of stone events, such as colic or expulsion of calculi (self presenting, annual x-rays), during the 5-year follow-up period. The study used 24-hour urine collections to determine the urine stone risk profile, including various urinary parameters. The study does not specify the method of random sequence generation or the process of distributing treatment assignments to participants. There is no information provided about the blinding status of the person allocating the random treatment assignments, patient blinding, or blinding of outcome assessors.

The study initially had 750 stone patients, and 220 of them were included in the study. However, the study does not report a power calculation and the rationale behind the chosen sample size. The statistical analyses used included analysis of variance (ANOVA) and Student’s t-test for unpaired data to assess differences between groups and at various follow-up times. A Chi-square test was used to compare the frequency of stone events. The level of significance (alpha) used for the statistical tests was set at p < 0.05.

## Summary of outcomes

The study included 220 patients, with 199 completing the study, comprising 99 in Group 1 and 100 in Group 2. The mean age in Group 1 was 42.2 ± 11.6 versus 40.4 ± 13.2 in Group 2. The male-to-female ratio was 70:29 in Group 1 versus 64:36 in Group 2. The mean body weight was 71.2 ± 11.6 kg in Group 1 versus 68.4 ± 13 kg in Group 2. Occupations with increased dehydration risk included tradesmen (15 versus 13) and farmers (4 versus 1). Occupations with reduced dehydration risk included managers (1 versus 2) and housewives (15 versus 20). During the 5-year follow-up, 11 dropouts occurred in group 1 and 10 in group 2.

To address question 1 – is baseline urine volume associated with stone formation – a community sample of patients was used as a comparator. The baseline characteristics, including age, sex, body weight, and type of working activity, were similar between groups. However, the baseline urine volume was significantly lower in male and female patients with stones compared to the control population (Male controls 1,401 ± 562 m1./24 hour versus male patients 1,057 ± 238, *p* < 0.0001; female controls 1,239 ± 440 versus female patients 990 ± 230, *p* < 0.0001).

In the 5-year follow-up period, 12 out of 99 patients in protocol 1 and 27 out of 100 patients in protocol 2 experienced a second episode of calculus. The recurrence rate was significantly different between the two treatment protocols (12.1% vs. 27% *p* = 0.008),. Additionally, the mean time to recurrence was significantly longer in protocol 1 (38.7 ± 13.2 months) compared to protocol 2 (25 ± 16.4 months) (*p* = 0.016).

Urine volume and supersaturation of lithogenous salts (calcium oxalate, brushite, acid, uric acid), which were similar in both groups of stone formers at baseline, showed significant differences at each annual visit during the 5-year follow-up. At 1 year, there were significant differences in the relative supersaturation of both Calcium Oxalate (5.2 ± 3.20) vs. (8.1 ± 5.3) (*P* < 0.0001) and Uric Acid (1.72 ± 1.49) vs. (2.66 ± 2.3) (*P* < 0.001) between protocols. At the 2-year follow up the measurements for Calcium Oxalate (4.4 ± 2.9) vs. (9.5 ± 5.2) (*P* < 0.0001), Brushite (0.84 ± 0.72) vs. (1.33 ± 0.72) (*P* < 0.0001), and Uric acid (1.29 ± 1.19) vs. (3.02 ± 2.72) (*P* < 0.0001) showed universal significance in relative supersaturation levels. Lastly, at the At the 5-year follow up the measurements for Calcium Oxalate (2.6 ± 0.8) vs. (9.9 ± 3.4) (*P* < 0.0001), Brushite (0.48 ± 0.24) vs. (1.58 ± 0.99) (*P* < 0.0001), and Uric acid (0.66 ± 0.35) vs. (3.46 ± 3.31) continued to report significance difference in supersaturation levels. Urine volume was increased in the intervention group. Initially, the baseline urine for both groups of stone formers was distinctly oversaturated compared to normal subjects. However, during the follow-up period, the oversaturation of group 1 decreased to normal levels. Values for the relative supersaturations in 101 controls were reported as 5.87 ± 4.1 for calcium oxalate, 0.83 ± 0.73 for brushite, and 2.65 ± 2.19 for uric acid.

Comparison of baseline stone risk parameters between patients with and without recurrences in both groups showed that baseline urine for patients with recurrences contained larger quantities of calcium in both groups (Calcium quantities (mg./24hrs) Group 1 – no relapse: 233 ± 100, relapse: 326 ± 140; Group 2 – no relapse: 249 ± 107, relapse: 313 ± 113). However, no significant differences were observed in the other parameters examined. The study does not report any adverse events observed during the study.

### Assessment of evidence

The study’s materials and methods demonstrate several strengths in its design and approach. A uniform study protocol was applied to all referred stone patients since 1986. The study involved a thorough evaluation process, with patients being hospitalised for renal colic, receiving appropriate medical treatment, and undergoing various clinical, laboratory, and radiological assessments to identify potential causes of stone formation. Additionally, patients were followed as outpatients and subjected to shock wave lithotripsy or other urological procedures when necessary. The study’s initial sample size was substantial, including 750 stone patients, of whom 220 were deemed suitable for the study. The inclusion criteria ensured homogeneity, with patients having the first episode of idiopathic calcium nephrolithiasis and no other retained calculi or metabolic pathologies requiring dietary measures or drug therapy.

The study implemented a prospective design with a five year follow-up. The control group comprised 101 healthy volunteers who were well-matched with the stone patients in various demographic characteristics (the healthy controls were used to assess risk of initial stone formation, while the intervention assessed stone recurrence in those who had developed a stone already). Randomisation and an adequate control group help reduce selection bias and enhance the comparability of the treatment groups. The long 5-year follow-up period allowed for the assessment of long-term outcomes, such as recurrence rates and time to recurrence, providing valuable insights into the effectiveness of the treatment protocols.

Despite these strengths, the study also exhibits certain limitations that warrant consideration. There is no power calculation to justify the selected sample size, making it challenging to assess the study’s ability to detect meaningful differences between the treatment protocols. Limited generalisability is another concern, given that the study was conducted within a specific geographical area (Parma, Italy) which may restrict the applicability of the findings to other populations or regions.

The study does not provide information about adverse events, which is crucial for evaluating patient safety and the overall risk-benefit profile of the treatment protocols. The paper mentions risks relating to salt imbalance but the absence of reported adverse events raises questions about the potential risks associated with the interventions. Additionally, the report does not disclose any potential conflicts of interest among the study authors or researchers, which could influence the study’s objectivity and interpretation of results. Many of these limitations reflect the era in which the study was conducted with current guidelines (e.g. CONSORT [7]) providing a more robust framework for the design, execution and reporting of randomised controlled trials.

Concerning bias, a 2020 Cochrane review analysed the study using the Cohrane’s ‘Risk of bias’ assessment tool, and reported a high risk of performance bias concerning the blinding of participants and personell [7]. The authors reported an unclear risk in the random sequence generation, allocation concealment, and blinding of outcome assessment, leading to potential risks in selection and detection bias [7]. Lastly the authors highlighted the missing outcome data in both protocol 1 (11/110 participants), and protocol 2 (10/110) resulting in an attrition rate of 10% and an unclear attrition bias [7]. 

## Future research

A Cochrane review published in 2020 identified this study as the only RCT assessing high water volume intake for secondary prevention of stone formation [8]. No RCTs were identified for primary stone prevention. Given the ongoing clinical need in this area, and increasing risk factors such as obesity (tripling between 1975 and 2016 [9]) there is a need for contemporary trials in this area potentially investigating community interventions to reduce the health burden of kidney stones.

## Data Availability

Not applicable.
